# Dual Caregivers: Stress, Physical Strain, and Self-Rated Health

**DOI:** 10.1093/geroni/igaf122.2355

**Published:** 2025-12-31

**Authors:** Sarah Clem, Stephanie McCorvey, Joan Davitt

**Affiliations:** University of Maryland School of Social Work, La Junta, Colorado, United States; University of Maryland at Baltimore, Baltimore, Maryland, United States; University of Maryland, Baltimore County, Baltimore, Maryland, United States

## Abstract

While research on the “sandwich generation” emphasizes the impact of simultaneously caregiving for both an older parent and a child, other contexts of dual caregiving require further exploration. Relying on a convenience sample of caregivers to vulnerable adults in one east coast state, this study examined caregiving stress, physical strain, and self-rated health between caregivers of one person (n = 1,161) and dual caregivers. In addition to caregiving for a vulnerable adult, dual caregivers included in the study were: a) those who also care for a child under the age of 18 (n = 364), b) those caring for another adult with a developmental disability (n = 151), and c) those caring for another older adult (n = 427). Associations were examined using ordered logistic regression, controlling for race, gender, caregiver age, caregiving difficulty, and service utilization. Dual caregivers with a child under the age of 18 had 55% (p<.01) greater odds of caregiving stress, 106% greater odds of increased physical strain (p<.001), and 94% greater odds of poorer self-rated health (p<.001) compared to caregivers for an individual adult. Similarly, the odds of poorer self-rated health were also greater for those caring for another older adult (OR = 1.68, p<.05) or another adult with a developmental disability (OR = 1.61, p<.01). Though, no associations were identified between these two types of dual caregivers compared to caregivers of a single vulnerable adult when exploring stress and physical strain. Results highlight how different contexts of dual caregiving impact negative indicators of caregiver wellbeing. Implications for systems supporting caregivers will be discussed.

